# Severe Ulcerative Colitis as a Complication of Mild COVID-19 Infection in a Vaccinated Patient

**DOI:** 10.7759/cureus.25783

**Published:** 2022-06-09

**Authors:** Maria Camila Fonseca Mora, Ashraf Abushahin, Rohit Gupta, Harry Winters, Gulam M Khan

**Affiliations:** 1 Department of Internal Medicine, Woodhull Medical Center, Brooklyn, USA; 2 Department of Gastroenterology and Hepatology, New York Medical College, Metropolitan Hospital Center, New York, USA; 3 Department of Gastroenterology, Woodhull Medical Center, Brooklyn, USA

**Keywords:** malnutrition, elderly, bloody diarrhea, covid-19 vaccine, covid-19, severe ulcerative colitis

## Abstract

Prior studies have proposed a direct correlation between the severity of coronavirus disease 2019 (COVID-19) and the severity of ulcerative colitis (UC). This is explained by an autoimmune response from molecular mimicry or via angiotensin-converting enzyme 2 receptor. However, we present a novel case of an inverse correlation between asymptomatic COVID-19 causing severe UC. An 84-year-old male with prior infectious colitis and asymptomatic COVID-19 presented with septic shock secondary to presumed infectious colitis. Blood workup suggested inflammatory bowel disease, which was confirmed to be UC via flexible sigmoidoscopy and pathology. He was managed successfully with oral mesalamine and high-dose intravenous steroids, which were later transitioned to an oral taper. This case reflects that the intensity of the impact of COVID-19 on the gastrointestinal system can be as variable as the immune system reaction in an already labile environment such as in the elderly and malnourished patients. Therefore, in view of a lack of reports establishing a relationship between these two entities, we aim to report a case and propose an association between mild or asymptomatic COVID-19 and severe UC.

## Introduction

Ulcerative colitis (UC) has been proven to have an economic and physical burden upon patients, with an associated mortality of up to 2.9% [1]. However, morbidity and mortality [2] have peaked since the breakout of the coronavirus disease 2019 (COVID-19) pandemic as result of the direct relationship between inflammatory bowel disease (IBD) severity and adverse outcome from the severe acute respiratory syndrome coronavirus 2 (SARS-CoV-2) infection [1].

The aforementioned virus has been reported to colonize the gastrointestinal tract and cause diarrhea in 31% of patients with COVID-19 pneumonia, even after respiratory symptoms have resolved [2]. This could be related to an autoimmune response via molecular mimicry [3] or via angiotensin-converting enzyme 2 (ACE-2) receptor [4].

Despite a prior proposal of direct correlation between these two entities in the past, available reports have failed to establish a relationship between mild or asymptomatic COVID-19 and severe UC.

The aim of this paper is to report the case of a patient with an asymptomatic pulmonary COVID-19 infection that had reactivated a quiescent UC [2,3] to its highest stage of severity.

## Case presentation

An 84-year-old Hispanic male with a history of bullous emphysematous chronic obstructive pulmonary disease status post-video-assisted thoracic surgery for recurrent pneumothorax, essential hypertension, type 2 diabetes mellitus, abdominal aortic aneurysm, and prior infectious colitis who presented in December 2021 for recurrent diarrhea and dysphagia. He had no upper respiratory symptoms but was incidentally found to be COVID-19 positive. He had two doses of Moderna vaccine, with the last dose eight months prior to presentation. The patient mentioned that in the past he had intermittent episodes of watery diarrhea that had self-resolved without intervention; however, during this presentation, he had bloody diarrhea intermittently for the past month with sporadic two to three bowel movements per day. This was associated with a 30-pound weight loss, poor appetite, and generalized abdominal pain. Computerized tomography (CT) scan prior to admission showed diverticulitis with mild colonic inflammation. He never had a prior endoscopy or colonoscopy.

He was initially admitted to the intensive care unit for septic shock secondary to COVID-19 versus severe colitis requiring norepinephrine for two days, fluid challenge with crystalloids, and broad-spectrum antibiotic coverage with vancomycin and Zosyn. His course was complicated by atrial fibrillation with a rapid ventricular response and persistent diarrhea which required modification of antibiotics to ciprofloxacin and metronidazole. Eventually, the patient was stabilized and transferred to the regular floor, where a workup of his diarrhea demonstrated positive fecal leukocytes and elevated calprotectin of 422 µg/g (reference: 0-120 µg/g) with a repeat of 744 µg/g. Other stool samples including elastase, gastrointestinal panel polymerase chain reaction (PCR), and stool fat were negative. One week later, a rectal tube was placed to accurately monitor stool output; additionally, he was placed nil per oral to better distinguish secretory versus osmotic diarrhea given that the prior osmolar gap was suggestive of a secretory etiology. At this time, a new diarrhea workup was sent; results were consistent with negative *Clostridium difficile* panel and ova and parasites. However, fecal leukocytes were positive, fecal lactoferrin was elevated at 277.93 (reference: 0-7.24), and calprotectin was 3,160 µg/g. Given the significantly elevated calprotectin and fecal lactoferrin, there was a high suspicion of intestinal inflammation likely related to IBD.

**Figure 1:**
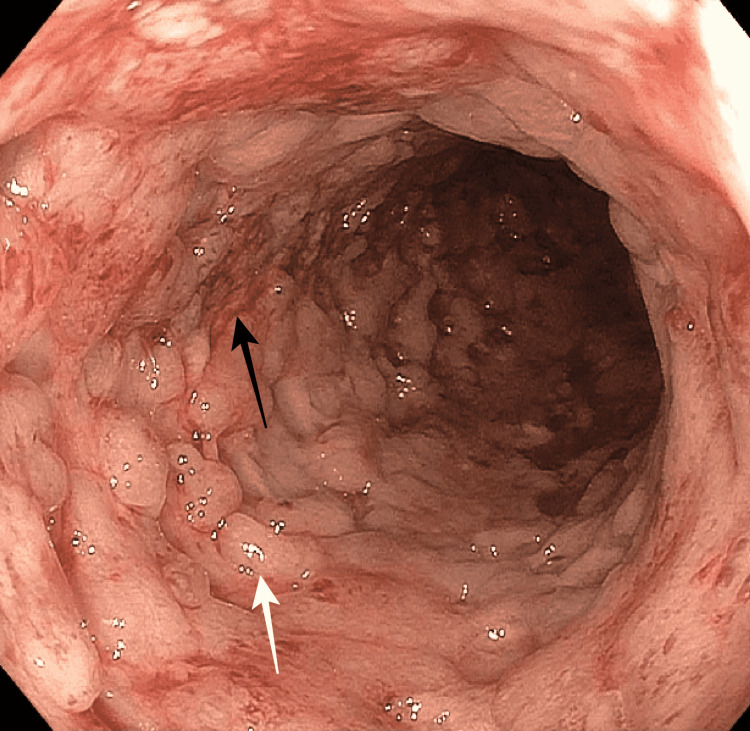
Cobblestone appearance at the descending colon. The black arrow demonstrates erosions and erythema and the white arrow demonstrates mucosal congestion.

**Figure 2:**
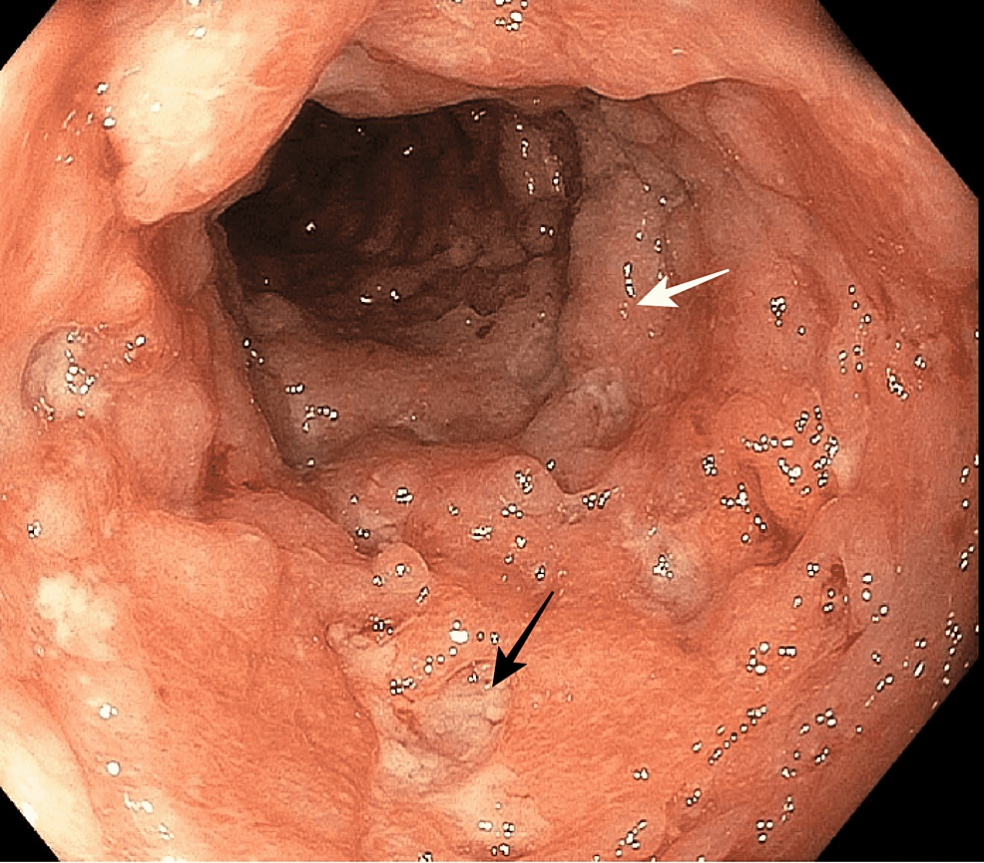
Cobblestone appearance at the sigmoid colon. The black arrow demonstrates deep ulcerations and the white arrow demonstrates mucosal edema.

Following hospital protocol, once he completed 10 days of isolation due to the diagnosis of COVID-19, we proceeded to perform a diagnostic colonoscopy. In January 2022, the patient underwent a flexible sigmoidoscopy with evidence of severe continuous inflammation given by mucosal edema, erythema, and mucous with multiple ulcerations and erosions extending from the rectum up to the descending colon (Figures 1, 2). There was no evidence of bleeding. Biopsies were taken from the descending colon, sigmoid, and rectum. At this point, we recommended ciprofloxacin 500 mg Intravenously twice a day (BID) and metronidazole 500 mg intravenously three times a day (TID).

**Figure 3:**
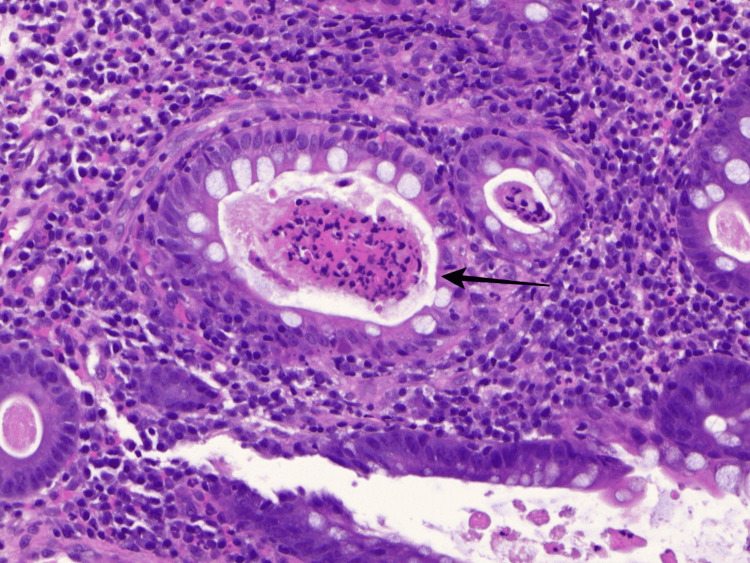
Pathology slides from descending colon biopsy displaying a cryptal abscess. The black arrow demonstrates neutrophil infiltration at the lumen of the crypt suggestive of a cryptal abscess.

**Figure 4:**
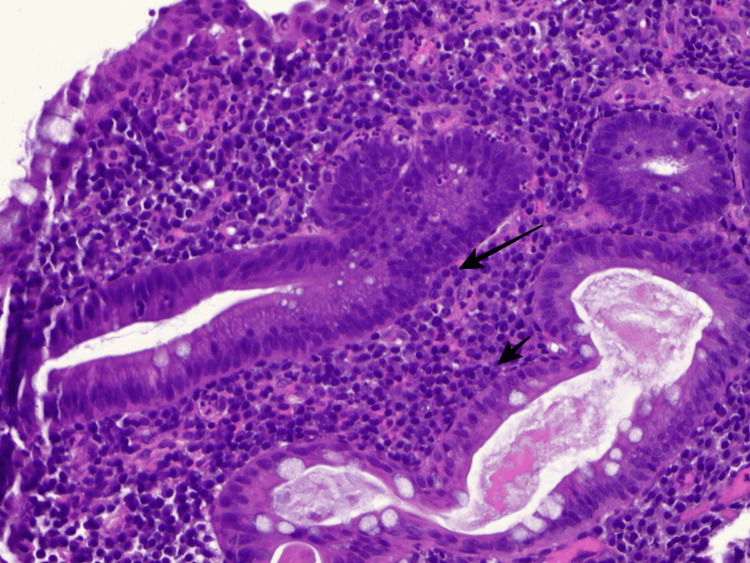
Pathology slides from descending colon biopsy consistent with cryptitis. Black arrows demonstrate multiple neutrophils in the crypt epithelium consistent with cryptitis.

**Figure 5:**
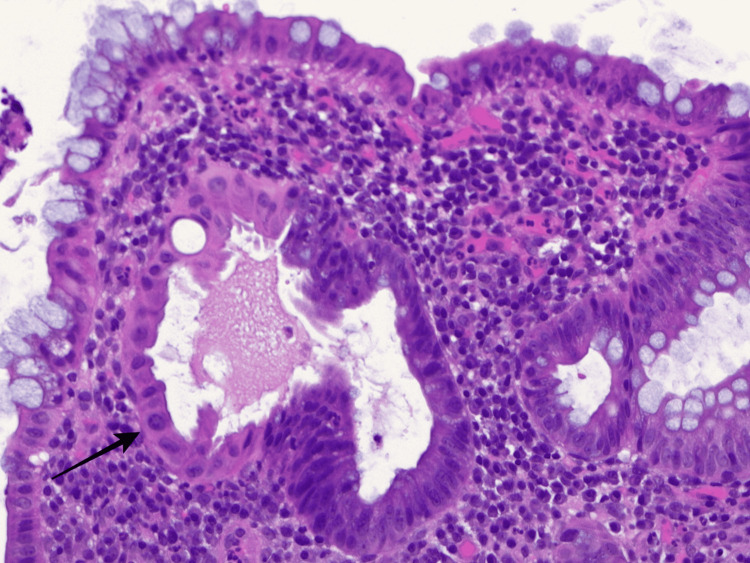
Pathology slides from descending colon biopsy displaying a cryptal arch distortion. The black arrow demonstrates loss of structure of the crypt arch.

Two days later, pathology demonstrated severe acute and chronic inflammation, focal cryptitis with crypt abscess (Figures 3, 4), granulation tissue formation, crypt architectural distortion (Figure 5), and reactive and regenerative epithelial changes from the rectum to the descending colon consistent with UC. Given the aforementioned, the patient was started on methylprednisolone 20 mg intravenously TID and oral mesalamine daily as per guidelines, and antibiotics were discontinued. Five days after initiation of treatment, we repeated calprotectin and fecal leukocytes which were 560 µg/g and negative, respectively. Methylprednisolone was transitioned to prednisone 40 mg daily to begin a tapering dose. Currently, the patient has improved diarrheal episodes (off psyllium) without recurrence of bloody events. He was also able to resume oral intake after previously being managed with peripheral parenteral nutrition.

## Discussion

Gastrointestinal manifestations secondary to SARS-CoV-2 infection are now considered one of the most frequent complications of this disease, both during the active infection and up to five months after treatment of COVID-19 pneumonia [2]. There is still minimal data on ongoing gastrointestinal syndromes in asymptomatic patients with mild COVID-19 infection. The development of the aforementioned pathology is thought to be related to persistently elevated ACE-2 activity in the terminal ileum, colonic mucosa, and lungs, explaining the frequent association between COVID-19 pneumonia and diarrhea [4]. Through microarray transcriptomics, multiple studies have shown increased expression of ACE-2 RNA in UC among 70% of patients versus 30% of Crohn’s disease patients. Additionally, the latter decreases the expression of tryptophan, leading to niacin deficiency and subsequent increased intestinal inflammation [4].

Since the outbreak of the pandemic in 2019, four de-novo cases of UC have been reported related to COVID-19 infection. However, only one case was reported without prior pneumonia. Early on in the pandemic, Calabrese et al. reported a case of a 19-year-old female who presented with fever, vomiting, bloody diarrhea, and loss of taste. CT was only significant for enhanced ileum and colon, but no lung pathology. Gastrointestinal symptoms persisted despite treatment with hydroxychloroquine; therefore, biopsies were done which were suggestive of UC [5]. Similar to our patient, no respiratory symptoms were evident prior to developing IBD; however, our patient was elderly with a prior history of mild infectious colitis [3,4], implying that his colon had a high risk of clearing normal gut flora. This, in turn, is worsened with SARS-CoV-2 as it modifies the gut microbiota leading to a dysregulation of the normal flora bacteria, as well as increased inflammation from the virus itself by angiotensin II overexpression [4,6]. All this can suggest a possible cause of reactivation or de novo UC in our patient.

The remaining three cases were preceded by COVID-19 pneumonia in the absence of known UC. The average age was 52.5 years (50-55 years), 66.6% were males, and, in all cases, the initial diagnosis of UC was foreseen until failure to improve with COVID-19 treatment as its presentation seemed to be unlikely related to UC. Stool calprotectin [3,4] was sent in all cases, with only one reported value of 1800 mg/g (reference 0-120 µg/g). All of the cases were confirmed with colonoscopy and tissue biopsy, and only one group used additional endoscopic ultrasound to diagnose de novo UC at the sigmoid colon [6]. After confirmation of UC, patients were treated with oral and/or topical mesalamine (oral and topical) [2,4,6,7]. Similar to our case, a detailed diarrhea workup was done after the management of other medical pathologies; moreover, our patient did not have a prior diagnosis by biopsy or endoscopy. The fact that COVID-19 can trigger autoimmunity leaves an open question in this case. It is known that the virus generates molecular mimicry, activating immune response to antigenic epitopes distinct from the disease-causing epitopes, activating T cells by bystander activation, or exposing cryptic epitopes [3]. This might have been our patient’s scenario as he did not have UC manifestations before but mild colitis that then worsened into severe bloody diarrhea with high inflammatory markers such as calprotectin more than 3,000 µg/g. It remains unclear if he had a mild undiagnosed UC in the past that became a flare or if this was a de novo infection; however, we do know that COVID-19 infection had a strong impact on the decline of his health.

Given that our patient had a lengthy and mostly atypical presentation compared to the cases that have been described, we also considered the possibility of underdiagnosed UC that had now flared as a result of COVID-19 infection. Venkatachalam et al. reported the case of a 31-year-old female with a history of mild UC on mesalamine who presented with abdominal pain and diarrhea for 10 days associated with COVID-19 pneumonia. Both the elevated calprotectin and positive CT findings suggested a flare, which upon subsequent sigmoidoscopy revealed a moderate-to-severe UC [3]. As with our patient, due to concerns of intra-abdominal pathology, the patient received ciprofloxacin and metronidazole until the confirmatory findings via biopsy, and they were further treated with Solu-Medrol. However, as opposed to our case, this patient had severe COVID-19 which also highlights the uniqueness of our case as the first asymptomatic COVID-19 case with severe UC.

It has been hypothesized that an already liable intestine is prone to any infection, and COVID-19 is not an exception. Mucosal breakdowns from prior infections in addition to the baseline elevated levels of ACE-2 and transmembrane serine proteases in the gut are the perfect mix for the SARS-CoV-2 virus to enter the cells and release an inflammatory cascade severe enough to either reactivate dormant UC or “generate” a de novo autoimmune response in a genetically predisposed patient [2]. Additionally, the fact that our patient had a prior vaccination a few months before developing colitis raises the question if the mRNA present in the vaccine might have been related to the progression of the disease.

## Conclusions

The impact of SARS-CoV-2 is certainly beyond respiratory syndrome. Even in patients who are asymptomatic for airway issues, gastrointestinal compromise should remain as a high priority, especially in patients who have high risk factors such as malnutrition or prior mild colitis events.

Mild COVID-19 can be a cause of reactivation or flare of severe UC as the gastrointestinal tract is already labile and prone to the proinflammatory effect of the virus; it can be substantial enough to cause an aggravating presentation.
